# Domestic

**Published:** 1840-10-31

**Authors:** 


### DOMESTIC.

*Medical Department of Yale College.*—The session of this institution for 1840–41, is attended by fifty-two students—an unusually large number.

---

The subjoined remarks from the Boston Medical and Surgical Journal, are deserving the attention of our readers. To physicians wishing to dispose of, or purchase a practice, the advertising sheet of the Examiner, with its large country circulation, will be an excellent medium of making their desires known.

*Purchasing a Practice.*—Gentlemen in pursuit of a location to practise, are becoming a numerous body. It being quite impossible to find an unoccupied place, the prominent object of solicitude is to ascertain who is in want of a partner, or who is desirous of relinquishing business. It has been found, by experience, that the shortest way of doing this, is to speak through the medical journals. A single line, in the form of an advertisement, will accomplish more for the advertiser in one week, than a whole year devoted to travel in search of a promising residence—and hence the custom of advertising by those wishing to dispose of their practice, as well as those who wish to procure one, is becoming very general. In London there is a class of medical brokers, exclusively devoted to negotiating between persons of this description. The same custom may at some future time prevail here. As a general rule, an objection is made to purchasing the house, fixtures, and the like, which the seller may happen to possess, and which would be as useful to the purchaser as they were to the owner, in pursuing the same professional routine. Again, in most instances, there is an unwillingness to pay a bonus for the good will or patronage of the occupant. Both of these objections are generally wrong. If a stranger can step at once into the custom of a physician, who, perhaps, may have been twenty years in obtaining the confidence of the community, it is right and proper that he should pay for it; it is economy to do it, since he is thus introduced to the patrons of the seller, whose ability to put him into the immediate receipt of a substantial income, it is presumed has been first ascertained.

We are quite confident that young physicians would be gainers, as a general rule, by purchasing the stand of a practitioner of character. It is not difficult to ascertain every fact that it may be essential to know—and the cost should be proportioned to the extent of the field. Reference is here made to the country exclusively. To purchase a practice in a city would be ridiculous: a transfer could not be effected, constituted as society is and always must be in a dense population. In the country it is reasonable and judicious to buy and sell a medical patronage, and the expediency of the measure will ultimately, we believe, be universally admitted.

---

*Medical Society of Tennessee.*—This Society at its meeting in May offered a premium of fifty dollars for the best Essay on *Bilious Fever*, to be submitted under the following circumstances:

"Dissertations on this subject must be transmitted, post paid, to Samuel Hogg, M. D., Nashville, Tennessee, on or before the 1st Monday in March, 1841.

"Each Dissertation must be accompanied with a sealed packet, on which shall be written some device or sentence, and within shall be endorsed the author's name and place of residence. The same device or sentence is to be written on the Dissertation to which the packet is attached.

"All unsuccessful dissertations are deposited with the Corresponding Secretary of the Society, from whom they may be obtained if called for within one year after they have been received."

The Committee appointed to read the Dissertations and award the prize, consists of Drs. Felix Robertson, Thomas R. Jennings, and J. H. Atkinson, of Nashville.—*Western Jour. of Med. and Surg., Nov.* 1840.

---

*Intermittent Fever, Hepatitis, Pneumonitis, Laryngitis and Intestinal Mucous Irritation.* By Daniel Drake, M. D., Professor of Clinical Medicine and Pathological Anatomy in the Medical Institute of Louisville.—We have written down these words to draw the reader's attention to a case which this day fell under our observation. We do not claim for it any *very* remarkable character, but as it is perhaps equal in interest to much that is published in medical journals we venture to send it forth.

Edward Dykes, a labouring man, living in the valley of Salt Creek, Chillicothe, Ohio, aged 32, of a sanguine temperament, and free from tubercular or other hereditary taint, was attacked about sixteen years ago, in autumn, with ague and fever, which lasted two months. For the next fourteen years it returned almost every autumn. During that long period, he continued most of the year to labour, and was free from cough, but sometimes had irritable bowels with slight diarrhœa.

In August, 1838, he was seized with the malady which had so often attacked him before. The type was at first tertian, and the cold stage amounted to a shake; at length became quotidian, with a chill only, about six o'clock, P. M. He continued to go about. Living with a "steam-doctor," he took two profuse sweats, and after the second "caught cold," and began to cough; at first without pain, but at length that symptom set in—being "located" in the right hypochondrium. His expectoration was sparing. He took two vomits of ipecac. but was neither bled nor blistered. Throughout the whole of the following winter, 1838, '39 he continued to have an evening chill, followed by fever in the early, and sweats in the latter part of the night, and frequent attacks of diarrhœa. His cough still continued with little expectoration. His larynx became somewhat sore and his voice and cough hoarse and flat, which sounds have continued ever since. Much eating brought on fits of coughing, and these brought up, by exciting a retrograde œsophageal action, the food which oppressed his stomach. Throughout the whole of the year 1839, these symptoms, including the evening chill, continued to return upon him, but he still kept on his feet, and did not become very much emaciated. In the month of January last, 1840, he awoke in the morning with a severe pain extending from the clavicle to the short ribs on the right side, with sense of suffocation, and a new reduction of the powers of his voice. He was bled, cupped, blistered, and took expectorants; under which the symptoms rapidly abated. Since that time he has not had evening chills, nor morning sweats; though he thinks his feet and hands hot at night; but his diarrhœa has continued. He has from five to eight evacuations daily. The discharges in the forenoon, often appearing to have received a tinge from the food he had taken the day before, but frequently of a light hue. In the afternoon they are small in quantity and attended with tenesmus, and very commonly have the appearance of "matter from a sore," which has been the case for eighteen months. His urine is often saffron colored. His cough is slight and his expectoration extremely limited. Twice or thrice lately the mucus has been tinged with blood.

At this time he has a moderately furred tongue, natural in size and colour. His eyes, although a little turbid, are not jaundiced. The skin generally is reduced in whiteness, but that of his face is dark and sallow, nearly up to his hair. The ends or balds of his fingers are a little enlarged. His pulse is 76, quiet and regular—not hard.

*Examination of the Neck and Chest.*—*Larynx* with a tender spot on the right side, within the thyroid cartilage. Os hyoides exceedingly moveable. A grating sound when the larynx is moved by the hand. *Chest*, when examined, naked and in quiet respiration, well formed, except a little bulging out of the most convex part of the false ribs, of the right side; which side, however, has the same circuit with the left both there and above. Breathing chiefly diaphragmatic. When directed to make a deep inspiration, his left shoulder ascends and the ribs rise and bulge out; but the right remains unmoved. When a cord was passed round the body, with its middle pressed upon one of the spinous processes, and the ends were brought to the sternum, a deep breath rendered the last extremity too short, but there was no recession of the right end. Under percussion no hollowness was discovered over any part of the right lung or of the *sternum*, either in the erect or recumbent position—the hollow sound of the left side was rather greater than common. Ausculation could detect no respiratory murmur, any where over the right side; but disclosed a considerable degree of broncophony, with an occasional sibilant rhonchus. His respiration throughout the left lung was puerile.

The position and movements of his heart were natural.

Percussion, pressure and deep inspirations excited very little cough.

*Examination of the Abdomen.*—No enlargement of the spleen or liver; no tenderness in the region of the former, but decided soreness in the latter, extending into the epigastrium. General soreness on pressure over the abdomen, but especially across it, in a zone passing a little below the umbilicus. Soreness in both iliac regions, extending upwards and backwards towards the hypochondriac and lumbar regions. Slight general fulness of the abdomen—more or less pain—and considerable flatulence.

*Examination of the Spine.*—No tenderness except near the junction of the vertebræ with the sternum. No tendency to cough from pressure.

For some time preferred to lie on his left side—now lies easier on his right; walks and rides about, and thinks himself much better than he was last winter.

The recurrence of ague and fever upon this patient, through such a long term of years, is not unprecedented, as such things have happened before in the alluvial valleys of the West; but the transition which occurred in the autumn of 1838, from miasmatic, to pseudo-hectic fever is a phenomenon not often seen. The tertian character and the shake of the annual attack of that autumn, seem clearly to characterize it as of malarious origin; and, moreover, he had at that time no cough nor other pulmonary difficulty, as far as we could gather from him. But at length he became affected with a double tertian or quotidian, and while this was still returning upon him, pneumonitis of a mild kind set in, and soon afterwards night sweats began, and with the chill and nocturnal fever continued for twelve or fourteen months; when in January, 1840, he seems to have had a re-inforcement of the pneumonic inflammation. Thus we have before us a series of little interruption, for eighteen months, commencing as an endemic *ague and fever* and ending as well marked *hectic*, with a transition from one to the other, in the most imperceptible manner. Still further, the fever assumed a hectic character, while the pulmonary affection was yet too recent, according to common experience, to have produced it. It appears from this case, that a miasmatic fever has not the power to ward off a hectic, notwithstanding that opinion has had its advocates. Did not indeed the existence of a chronic intermittent, at the time the pneumonitis set in, facilitate the developement of hectic—a paroxysmal fever with an evening chill, *naturally*, recurring at the same hour, which in him was the hour of recurrence of the miasmatic chill? Did not the two modes of constitutional morbid action, notwithstanding the difference in their origin, so much resemble each other as to coalesce? When, in singing, we open the mouth in the *manner* it is opened in yawning, that function is apt to be performed, although the condition which naturally prompts it is not present. But we shall not speculate further on this connexion, but request such of our readers as live in regions where ague is endemic, to keep on the look out for facts having a bearing on its relations with hectic fever.

What is the morbid anatomy of the lungs of this patient? The left is sound and sustains the *onus* of respiration. The right would appear to be impervious throughout, at least in its vesicular structure. The larger bronchial ramifications, only, admit air. How long it has been thus hepatized cannot be ascertained. From the general state of feeling of that side, and the condition of his pulse, it would appear that the inflammation had nearly ceased. Has the secretion into the parenchyma, been so great as to obliterate the capillary system to such an extent, that the inflammation ceased? May not a lung continue a long time in that condition, as it often does in a state of induration, from the compression of coagulable lymph, contracting around it? We have certainly met with cases of hepatized lung, connected with mild occasional hectic fever, which have continued for several years; and we may suppose that some very chronic cases which were regarded as tubercular have been of this kind.

The laryngeal affection in this case is distinctly marked. The relaxation of all the ligaments and muscles is indicated by the looseness of structure of that organ, and the play of the os hyoides upon it; not less than by the feebleness and flatness of the voice and cough. It is not impossible that the mucous membrane of the right side is in a state of superficial ulceration. The tinge of blood which he lately threw up, perhaps came from such a spot. Whatever may be the exact pathology of this organ, it is, perhaps, sympathetic of the disease in the right lung.

Let us turn our attention to the abdominal viscera. It is worthy of note, that notwithstanding the repetition of ague for many successive years, the spleen is not enlarged; and we may here say, what should have been said before—there is no anascarcous infiltration into the extremities.

The liver is undoubtedly in a state of chronic inflammation. The tenderness under pressure, the occasional saffron color of the urine, the turbid and sallow hue of the skin, the early tendency to diarrhœa, the tenderness *in epigastrio*, and the cough with regurgitation, after a full meal, may be received as evidence of disorder of the liver. The preference for lying on the right side is ambiguous, inasmuch as the great amount of respiratory duty thrown upon the left lung, requires that the weight of the body should not rest on that side. But what shall be said of the bulging out of the false ribs over the liver? Was there a swelling, inflammation and abscess of that organ, some time ago, which pressed out and changed the convexity of those ribs? Is the liver at this time enlarged *upward and outward?* Is there a collection of pus in the sac of the pleura, resting upon the diaphragm? If so, there must be adhesion of the membrane above, for the protuberance is as great in a recumbent, as upright posture.

At this time, and indeed, for many months, the more energetic morbid *actions* in this case, seem to be in the mucous membrane, more especially, perhaps, that of the great intestines. We did not see the discharges which the patient described as purulent, but presume they may be of that character. Are they from the liver, or from the intestinal membrane? The latter is most probable; and taken in connexion with the general abdominal tenderness, and the tenesmus, can leave little doubt of ulceration—probably in various places. Has this condition of the membrane arisen directly from the ague and fever; or is it a sympathetic effect of the liver disease; or has it come on in the way it arises in the progress of hectic fever from tubercle?

We shall conclude these off-hand pathological speculations with a few clinical remarks and suggestions.

It appears to us, that the right lung had passed through a transformation, and that although it will doubtless, at no distant time, be the seat of a revived and suppurative inflammation of a fatal kind, there was not any existing morbid action upon which remedies need to be directed, or, indeed, could be, if morbid *action* did exist. Our thoughts turned to the state of the liver, and especially the intestinal membrane, both of which, at the present moment, seem to be the seats of sub-acute inflammatory action. Although, however, we have called the action *inflammatory*, we cannot regard it of such a grade as to call for depletion. Moreover, there seem to be several objections to a depleting plan. 1st. The pulse is not hard nor frequent. 2d. An organic lesion, so extensive as that in the lungs, renders debilitating measures, to any degree, injurious. 3d. Ulceration of the bowels may be prevented by depletion, but it by no means follows that it can be cured by the same mode. Ulcers of the skin, of the vagina and urethra, of the meatus externus, of the conjunctiva, of the mouth and throat, especially when chronic, are not in general successfully treated with emollients and refrigerants, but with stimulants, astringents and escharotics, and often heal more kindly, if tonics be administered internally at the same time; wherefore should chronic ulcers of the colon and rectum be made an exception to these, and the patient subjected to a debilitating treatment? 4th. This patient for several weeks past, has been using a bitter tincture, made with whiskey, during which time there has been no aggravation of any of his symptoms, but an increase of flesh and strength.

Under these views we made the following prescription:—

1. A liquid, demulcent and gelatinous diet—in moderation.
2. The nitro-muriatic bath, to the region of the liver, epigastrium and umbilicus, every night.
3. A draught, three times a day, of ℥ij. of decoction of cinchona, and ℥ij. of lime water, mixed.
4. A pill every night, composed of two grains blue-mass, two of ipecac. and one of opium.

Are we asked, do you expect the final recovery of the patient under this or any other treatment? We answer no.

To such of our readers as may think their time lost by the perusal of this article, we beg leave to say, that the afternoon (July 29) is oppressively hot; and that, if we were writing in as pleasant weather as they will read, we should no doubt forestall them in that criticism. *Ibid.*

